# How can we establish more successful knowledge networks in developing countries? Lessons learnt from knowledge networks in Iran

**DOI:** 10.1186/1478-4505-12-63

**Published:** 2014-10-29

**Authors:** Bahareh Yazdizadeh, Reza Majdzadeh, Ali Alami, Sima Amrolalaei

**Affiliations:** Knowledge Utilization Research Center, Tehran University of Medical Science, No 1547, North Karegar St, Enghelab Ave, Tehran, Iran; Knowledge Utilization Research Center; Epidemiology and Biostatistics Department, School of Public Health, Tehran University of Medical Science, Tehran, Iran; Department of Health, School of Public Health; Social Determinants of Health Research Center, Gonabad University of Medical Sciences, Gonabad, Iran

**Keywords:** Knowledge network, Evaluation, Health management organization, Iran

## Abstract

**Background:**

Formal knowledge networks are considered among the solutions for strengthening knowledge translation and one of the elements of *innovative systems* in developing and developed countries. In the year 2000, knowledge networks were established in Iran’s health system to organize, lead, empower, and coordinate efforts made by health-related research centers in the country. Since the assessment of a knowledge network is one of the main requirements for its success, the current study was designed in two qualitative and quantitative sections to identify the strengths and weaknesses of the established knowledge networks and to assess their efficiency.

**Methods:**

In the qualitative section, semi-structured, in-depth interviews were held with network directors and secretaries. The interviews were analyzed through the *framework approach*. To analyze effectiveness, *social network analysis* approach was used. That is, by considering the networks’ research council members as ‘nodes’, and the numbers of their joint articles - before and after the network establishments - as ‘relations or ties’, indices of density, clique, and centrality were calculated for each network. In the qualitative section, non-transparency of management, lack of goals, administrative problems were among the most prevalent issues observed.

**Results:**

Currently, the most important challenges are the policies related to them and their management. In the quantitative section, we observed that density and clique indices had risen for some networks; however, the centrality index for the same networks was not as high. Consequently the attribution of density and clique indices to these networks was not possible.

**Conclusion:**

Therefore, consolidating and revising policies relevant to the networks and preparing a guide for establishing managing networks could prove helpful. To develop knowledge and technology in a country, networks need to solve the problems they face in management and governance. That is, the first step towards the realization of true knowledge networks in health system.

## Background

knowledge networks include a group of professional organizations that work on a common subject to strengthen each other’s research and communication capacities, share their knowledge base, and identify solutions which can satisfy national and international decision makers’ needs. The most significant advantage of *formal* knowledge networks, in comparison with other knowledge networks, is their influence on decision-making individuals and/or organizations [1]. Networks can act as bridges between research and policy [2]. On the other hand, doing research activities within a network and in the presence of a wide range of stakeholders is one of the approaches for strengthening the tie between knowledge producers and users in a *Knowledge Translation and Exchange* atmosphere [3]. The presence of knowledge stockholders in the knowledge networks pushes the data and information to produce the knowledge which will impact the decision of decision makers. Various factors influence the way networks are viewed. Among these factors are advances made in Information and Communication Technologies; recognition of the need for multiple interventions to solve social, economic and environmental problems; the disappointment of the community and academicians in the negligence of research findings in the decision makings process and; lack of credit and appreciation of the private sector’s experience [1]. Knowledge networks’ activities revolve around three main fundamental themes: joint projects and information exchange, interaction with stakeholders, and network management. Knowledge networks themselves are based on seven main principles [4]:

Knowledge networks

1. are purpose-driven
2. are working networks
3. require organizational commitment, in addition to the individuals’ and specialists’ participation,
4. are based on experience; not merely on personal or organizational interest
5. are cross-sectional and cross-regional
6. must empower and capacitate all the members, and finally
7. are interactive networks

Knowledge networks which are active in the field of health should move toward the goal of “better research for better health” by, first, supporting and leading research toward its highest quality and, second, setting up effective interactions with research centers [5].

In 2000, knowledge networks were introduced to Iran’s health system to organize, lead, empower, and coordinate efforts among medical research centers in the country. The statute passed for the establishment of the research networks included the following goals: determining national and macro-level research priorities in line with each network’s mission; coordinating and converging research themes among research centers; creating and strengthening the spirit of team-work among researchers and stakeholders; creating international collaborations with similar global research networks; promoting the quality and the quantity of research studies by making available data, resources, facilities, and equipment shared by other members of the network. These networks must, at all times, have at least one ongoing macro-level research project at hand with the collaboration of at least 50% of their members.

At the time this study was conducted, ten medical research networks were active throughout the country. These networks and their years of establishment (brought in parentheses) are as follows: Molecular Medicine (2000), Medical Biotechnology (2001), Medicinal Herbs (2002), Stem Cells and Pharmaceutical Sciences (2005), Traditional Medicine, Cancer, Neurology, Ophthalmology and Mental Health (2006), Infectious and Tropical Diseases (2007). On the whole, 204 research centers, schools and institutes had memberships in these networks.

Iran is among the countries that have had the fastest growth rate in the production of science in the past two decades in the world and in the region [6, 7]. Iran’s 20-year-perspective-document has been set for 2025. The country’s first approach toward achieving these visions is to prepare a long-term plan specific for advancing science and technology. The primary goal of this “roadmap” is to strengthen the national innovation systems; one of the components of which is knowledge networks [8]. An *innovation system* is defined as “an open network of organizations both interacting with each other and operating within framework conditions that regulate their activities and interactions”. Of the components of innovation systems are innovative activities and framework conditions (rule of game) [9].

The lessons learnt from establishing and maintaining knowledge networks in Iran can not only prove beneficial to improving the performance of knowledge networks, but also they provide insights into the status of innovation systems in developing countries. The current study was, therefore, designed to examine the experiences gained from the establishment of official knowledge networks in Iran. The results of this study will also be helpful for low- and middle-income countries with similar conditions of scientific and technological development, which their main research funds are publicly provided.

## Methods

As stated above, this study was conducted in two sections: qualitative and quantitative. The qualitative section examined the strengths and weaknesses of networks in their formation and performance; the quantitative section, on the other hand, was conducted by holding in-depth interviews with administrators and secretaries of the networks through *network analysis* procedures.

### a) Qualitative section

In-depth interviews were held with network directors and secretaries at their workplaces. The interviews began with questions like the following:

- “With what objective was this network established?”
- “Has the network achieved the outlined goals?”
- “Which obstacles have affected the network’s performance?”

The interviews were then continued based on the responses given to these questions. All interviews were audio-recorded upon the participants’ consent and subsequently transcribed verbatim.

The *framework approach* was used to analyze the qualitative section’s data. That is, after the initial *open coding* was done, the extracted codes were classified according to the themes proposed by the ‘Knowledge Networks: Guidelines for Assessment’ tool [10]. This tool was published by the International Institute of Sustainable Development in 2004. It introduced four categories of Effectiveness**,** Structure and Governance, Efficiency, and Resources for the assessment of networks. According to this guideline, network ‘effectiveness’ is defined as transparency of the network goals and the rate by which these goals are achieved according to the network’s strategic plan. The strategic plan elaborates the relations of network members with each other and with other decision makers who are influenced by the knowledge produced by the network (who), the relevant knowledge gaps (what), how the stakeholders are contacted (how), and eventually the decisions influenced by the knowledge produced in the network. In the ‘structure and governance’ domain, the following were assessed: network formation, network structure, and finally network formalizing. In the ‘efficiency’ domain, relations and interaction among members, organizational support, procedures and trends for determining network’s capacity for effective collaborations are assessed. In the ‘resource’ domain, human resources, financial resources, time and sustainability were evaluated.

Because no intervention was done in this study and it did not have any maleficent consequences for individuals and/or their personal lives, oral consent alone was obtained from its participants. The study has been approved by the Institutional Review Board of University of Tehran’s Medical Science Faculty which follows the Helsinki Declaration.

### b) Quantitative section

The goal of the quantitative section is to assess the effectiveness of the networks using the *social network analysis* method. This method has been suggested for evaluating member-participation in network activities and is used as an indicator of network effectiveness. In fact, social networks are comprised of a group of members which are connected to each other through meaningful social relations. These relations can be used to analyze the structures that have been formed by the members [11]. ‘Centrality’, ‘density’ and ‘nature and strength of relationships’ are among the indicators that have been suggested for this purpose [12]. ‘Centrality’ is the concept from that of a property of a single actor to that of a group of actors within the network [13]. ‘Density’ is expression of the total number of links present in relation to the total number of links that are theoretically possible for any given network. The measure of density is the number of links divided by the total possible number of links [14]. ‘Tie strength’ describes strength of the relationship between two people, and trust are two relationship features that have great impact on what happens in a social network [15]. Also another indicator **‘**Clique’, which is suggested in social network analysis, represents the internal coalition of a network [16]. In this study we used centrality, density and clique to describe the knowledge networks.

Bearing in mind the executive limitations of the project (e.g. recognition of articles attributed to the knowledge network’s projects in the database was not possible), it was decided that the ‘before and after uncontrolled’ method be used in the population which consisted of network research council members. The research council members were defined as ‘nodes’ and the numbers of shared articles with other network members were defined as ‘relations or ties’ between the nodes. The names of research council members were extracted from network documents. Then, their joint articles were searched in PubMed information bank. Two time spans were considered in this search; before the networks establishment (the number of years before the establishment of the network), and after the networks establishment (the number of years from the establishment of the network up to December 2011). These two time spans are equal, meaning whatever the number of years there are from the time of establishment of the network up to Dec 2011, the same number of years are considered from the time of establishment backwards. Hence, this period is not identical for all the networks. Next, the matrix was drawn and the numbers of joint articles in these two time spans were delineated. The goal of network analysis was to examine the difference between the numbers of joint articles published by research centers and individuals before and after network establishments. The data were analyzed by UCINET software.

## Results

### a) Qualitative section

In-depth interviews were held with ten network director and secretaries from the ten networks that had been established by the time this study was conducted. On the whole, the networks’ performances and their rates of achievement of their outlined goals were not desirable. One of the participant’s statements clearly reflects this matter:

“I will not give you the network reports… I will only report my colleagues’ and my own activities”.

The barriers mentioned in the interviews were classified into four main themes of ‘effectiveness, structure and governance, efficiency, and resources and sustainability’:

### ➢ Effectiveness

In this domain the changes in the production of knowledge, the steps taken to communicate with stakeholders, and the quality of the interactions were examined.

### Utilization of research results

Although there is a strategic plan in each network, there is no clear and definite plan for the practical utilization of research results. The results of the Infectious Diseases Network’s project, which is a member of ‘Center for Disease Control of the Ministry of Health and Medical Education’, are used more often because their research projects are ordered by the Center for Disease Control(CDC).

### Establishing network websites

Network websites are very important infrastructure for communication between stockholders. Based on the statements of the participants, the design and the updating of the network websites are not satisfactory. ,Therefore one of the network goals which was access to researchers’ information was not achieved either. One of the network secretary stated that some of the research centers did not give their information to the network secretariat; hence the network website was not up-to-date.

### ➢ Structure and governance

In this section, network management, network formation (member selection and duty assignments), network structure (management and member interactions and network leadership) and finally, network formalizing (procedures for decision making, preparations and implementation of a work plans) are evaluated.

### Governance

According to two of the participants, networks have not been *institutionalized* in the Ministry of Health; therefore, no clear responsibilities have been assigned for them either. At the moment, networks only have a general definition and their duties are not clear, so it’s not possible to specify precise evaluation criteria for them. Since their establishment, the objectives of networks have changed for many times and each time these changes have brought about a great deal of confusion. As stated by one participant, there have never been any specific goals and a suitable management system in the networks:

*“What bothers our networks is a lack of directionality. In fact, networks have no ultimate or long-term goals! Nor do they have any intermediate- or short-term goals! They’ve turned into research councils that receive 60 or 70 Tumans [Iran’s national currency] a year and only sit together in meetings and ratify projects.*

Participants had different views on the specialization of network activities in certain fields or diseases. A research deputy-manager of one of the network members believed that specific networks must be established for each disease or field in order enable networks to achieve their goals. On the contrary, the secretary of another network member thought that the general title of “research network” will be meaningless if networks were to work on a specific field, then instead of generally saying “research networks”, these institutions must be addressed as “disease X’s research web”. It must be noted that although networks are separated based on their areas of study, there are considerable overlaps among their field of interest.

Another problem is that, the formation of some organizations in the country, such as Headquarter of Innovation in Stem Cells, has brought the activities of similar research networks to a stop.

According to another participant: *“If only rules, regulations, bylaws, and all the policies were well-coordinated and arranged, just like a car’s gearbox, to work together and to do something big. In reality though, rules and regulations are not coordinated like a car, where all the bits and pieces are well in harmony. We work in one area, or an action begins, but then because the other gears and parts are not coordinated, the action comes to a halt; either the money’s not there, or the researcher isn’t interested, or I don’t know! The policies change rapidly. Numerous problems exist in this area.”*

In addition, the instability of the policies of the Ministry of Health on networks has complicated the situation for networks and has caused networks to function independent of the national administration policies.

Moreover, as stated by one of the network’s secretaries, the secretariats doe not have sufficient authority to lead and manage their responsibilities. One reason is the method by which funds are allocated to network projects. The network secretariats do not have a device to monitor the progress of the projects, because the allocated fund is directly deposited to the research centers’ accounts from the Ministry of Health and Medical Education (MOHME), so, the centers do not send their progress reports to the network secretariat.

### Network secretariat structure

The secretariats are either merged inside the MOHME or integrated within the research centers (i.e. they do not have a specific office or location), and have no space and human resources of their own. So, a major problem is carrying out daily activities and responsibilities in spite of shortage of human resources. In this study, because the secretariat of one such network was merged within the MOHME itself, funds could not be allocated to the network due to bureaucratic difficulties and the financial deficiencies that spur problems inside the network.

### Member selection

According to the participants, when network establishments began, members of research centers were chosen based solely on individual interests and abilities, and no specific criterion was considered. On the other hand, some research centers became network members only to benefit from its financial resources. Thus, this issue affects their level of participation in furthering networks goals. Additionally, the problems existent in networks have caused research centers to gradually lose their incentives to take part in network activities - the number of centers participating in strategic meetings is a proof of this matter.

### ➢ Efficiency

This section will inspect the network’s internal management and its relevant considerations.

### Interaction among the network secretariats and research centers and communications of research centers with each other

As stated by the interviewees, collaborations among the research centers are extremely weak. The significance of communication among the centers is so great that; because of communication weaknesses, one network could not be established at all.

### The centers’ incentives for entering the networks

At the moment, research centers gain points by joining networks. This kind of encouragement does inspire the idea of becoming a member, but does not create incentives for further cooperation with the network.

### ➢ Resources and sustainability

In this section, the adequacy of human and financial resources in networks will be examined.

### Financial resources

All the participants believed that the financial resources allocated to the projects are meager, and not in accord with the expectations of MOHME for presentation and administration of macro-level and whole-scale projects. It should also be noted that networks do not have an independent budget.

### Allocation of Funds to network projects

As stated above, funds allocated to research centers are not monitored by the network secretariat and making financial supervision is practically impossible. Many networks cannot grant the centers a new budget based on the developments in their projects.

### Administrative issues concerning the allocation of funds

The administrative process for fund allocation is as follows: The MOHME will deposit the fund to the bank account of research center’s university. It will then inform the network secretariat through an official letter. Afterwards, through another letter, the secretariat will let the research center know of the deposit. Only then, and after going through relevant procedures may the research center receive the allocated fund from the university. The big problem is the time it takes to inform the centers about their deposited funds (sometimes it takes up to 9 months). This is why in some cases the research center didn’t receive its fund from the university at all.

### b) Quantitative section

In this section, the numbers of joint articles published by the research council members of the 6 knowledge networks were extracted. This was achieved by considering the numbers of joint articles written in equal time spans before and after the network’s establishment (the number of years since the network’s establishment until December 2011 was calculated and equal number of years was considered for the time span before the network establishment).

**Figure 1:**
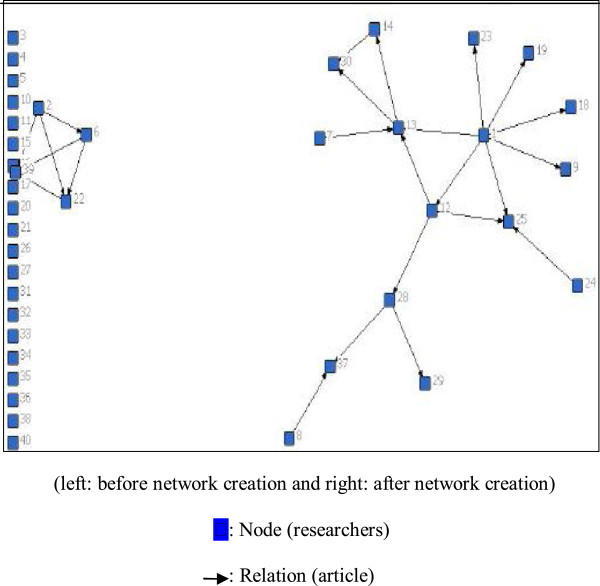
**Diagram of Knowledge network No 2.**

All articles extracted with this method have been listed in Table 1. The results of network analysis and its indicators are listed in Table 2. In these tables, the networks have been labeled with numbers 1 to 6. It must be noted that in the quantitative section, members of only 3 out of the 6 reviewed networks had joint articles both before and after the establishment of their corresponding networks. Figure 1 illustrates the network diagram for one the knowledge networks as example.

**Table 1:** **Number of individuals with joint articles and number of joint articles before and after the establishment of the networks**

		Number of individuals with joint articles		Number of joint articles	
Network number	Year of establishment	Before establishment	After establishment	Before establishment	After establishment
1	2001	2	4	5	27
2	2006	8	24	5	57
3	2005	10	17	14	170
4	2000	0	18	0	54
5	2005	0	6	0	9
6	2006	0	0	0	0

**Table 2:** **Network analysis indicators in each research network**

	Explanation												
		1		2		3		4		5		6	
Size	Number of researcher	13		41		35		30		20		5	
		Before	After	Before	After	Before	After	Before	After	Before	After	Before	After
Density	Mean (SD)	0.0642 (0.5625)	0.3462 (2.7166)	0.0061	0.0671 (0.5195)	0.0235 (0.1995)	0.2622 (3.0067)	----	0.1241 (0.8594)	----	0.0632 (0.4880)	---	0
Centralization	Percentage	8.33	8.14	4.62	6.50	9.57	5.72	----	6.65	---	4.68	---	0
Normalized degree centrality	Mean (SD)	1.282 (3.007)	1.442 (2.961)	0.610 (1.328)	0.958 (1.654)	0.784 (1.901)	0.546 (1.548)	----	0.955 (1.607)	---	1.053 (1.608)	---	0
Normalized closeness centrality	Mean (SD)	8.333 (0.000)	8.333 (0.000)	2.564 (0.064)	3.529 (0.450)	3.551 (0.009)	3.679 (0.330)	----	5.391 (0.057)	---	5.357 (0.133)	---	0
Normalized betweenness Centrality	Mean (SD)	0.000 (0.000)	0.000 (0.000)	0.013 (0.055)	0.644 (1.730)	0.112 (0.511)	0.117 (0.413)	----	0.854 (2.466)	---	0.029 (0.127)	---	0
Number of cliques	Mean (SD)	0	0	0	4	3	7	----	4	----	0	----	0

### Density

Since their establishment, Networks 1, 2 and 3, have experienced an increase in density. That is to say, the number of scientific interactions among the members of these networks has increased since their formation. Two of the networks (4 and 5) have joint article only after their establishment, so, the observed density only belongs to post-establishment period. Evidently, one network (Network 6) had no joint articles to imply the cooperation of its members before and after its formation. Even after its establishment, members did not produce a single research project.

### Centrality

After the establishment of Network 2, the highest centrality level was that of Member 1. This means Member 1 most likely enjoys a unique scientific, directorial or political status inside the network, and most published articles have been issued with this member's help and cooperation. After member 1, the most active participant is Member 13. The available information about this group indicates the formation of a subgroup, which consists of members 2, 6, 22, and 39. Among the members of Network 3, Member 15 shows the highest levels of centrality, while in Network 4, Member 2 held a similar position only after the network's formation.

### Clique

In social networking, cliques represent the internal coalition of a network. In this research, only cliques with a minimum of 3 members were examined. As illustrated in Table 2, Network 1 consisted of zero cliques both before and after its establishment; Network 3 had three and seven cliques before and after it establishment respectively, whereas Network 2 had zero and four clique before and after the network establishment.

## Discussion

Over time, network evaluation has proven itself to be a key element in the success of a knowledge network. The purpose of this study was to find the strengths and weaknesses of knowledge networks and to assess their effectiveness in Iran.

In the qualitative section it was determined that all four aspects including effectiveness**,** structure and governance, efficiency, and resources and sustainability had their own weaknesses. Non-transparency of the network goals and network management were among the most important problems. This weakness is reflected in the quantitative part. Network analysis results suggest that the mentioned knowledge networks cannot be considered *true networks* due to very limited interactions within them. This study reveals that after the establishment of networks, the number of joint articles of individuals has increased considerably. Apparently, the formation of knowledge networks in the country has provided a ground for further cooperation among researchers of the same discipline; However, when investigate more deeply, this proves not to be the case for two reasons. Firstly, the activities in the network are, according to a participant in the qualitative section, the result of individual interactions and incentives - not network interaction. Secondly, based on the density index, the number of members with joint articles in each network is very low; most of the collaborative articles only belong to a few members. In fact, internal coalition increased in only two of the networks (2 and 3) after their formation. As a matter of fact, even before its formation, members of Network 3 had already conducted joint studies which led to the publication of their results. The establishment of this network, most probably, created conditions which led to an increase in internal coalition of the members. On the contrary, within other networks, the establishment of a knowledge network has not been able to create internal coalition among members. As shown in other studies [17], the main weakness in Iran’s research centers is “centralized teamwork”, which makes them heavily dependent on the presence and status of certain individuals; hence their path to success is quite unstable.

A network's objectives should be determined at the very early stages of its establishment, as should be defined the path, assessment criteria, and the manner by which members will be selected [18]. Setting goals and specifying a problem (or problems) are among the key elements for establishing a new network. Without them, a recently formed network shall always remain in its infancy [19]. For example, if *capacitating* is the ultimate goal of a research network’s research and educational programs, then the research network must be homogenous. However, if the main objective of establishing a network is to *clear the path* from idea to implementation, then a heterogeneous set of stockholders (including basic, clinical and technological research centers, and pharmaceutical and medical companies, policy makers, patients) must be introduced into the network’s system.

Network management is about formalizing communications between members and being responsive to both members and non-members [20]. It is about how decisions are made about important issues within a network. It is the process through which organizations shape their interests, establish and realize their policies, manage differences and resources and fulfill their duties. Effective network governance is based on 5 principles: legitimacy, accountability, direction, performance, and fairness. It must be noted, though, each network can have different objectives, members, connections and values and a governing procedure may be designed and executed exclusively for each network [21]. Planning and monitoring tools must be designed to guide knowledge networks in these issues.

Knowledge network evaluation procedures are still very young. A number of methods have been proposed for knowledge network assessment including SWOT Analysis, Results Based Management, Logical Framework Analysis, Outcome Mapping, and Appreciative Inquiry. Each of these methods emphasizes their own criteria [22]. Nevertheless, to evaluate all the dimensions thoroughly, network objectives, its target audiences, and the level of assessment must be determined. For example, government- and community-based networks providing health services are advised to conduct the analysis in three levels: community, network, and organization/participant [23]. Furthermore, capacity building in a knowledge network occurs in three levels; individual, organizational and environmental levels, which may also be used for network evaluation [4]. From the knowledge networks’ audiences’ point of view, evaluation of a network where the members consist of caregivers, policymakers, and researchers, network analysis should cover two dimensions: network dimensions (network width, network components, and implementation site), and knowledge exchange dimension (exchange of knowledge, context of exchange, and facilities of exchange) [24]. And if the network’s main objective is to affect policies, then function evaluation is preferred to structure evaluation [25]. One of the latest approaches to knowledge network evaluation was introduced by Brian W. et al. They suggest three dimensions for network evaluation: Logic framework which is the same as the *input–output model*, and is especially useful once the knowledge networks set capacity building as its goal; Governance analysis which is the classification introduced by IISD (which was used in this study),but whose categories have changed to Resource, Structure and Governance, Progress Markers (IISD equivalent of ‘Efficiency’), Impacts (IISD equivalent of ‘Effectiveness’) and Sustainability; and finally, Network analysis, which stresses network evaluation based on the community of participants.

The writers of this report believe that network analysis examines the effects of the practices of a network on research centers and institutes. The main objective of network analysis is to find out the answer to a simple question: Has an actual network been formed? [26]. All network analysis methods focus on a knowledge network’s management and formation. A useful method to evaluate network impact is *payback framework*. A payback framework studies the impact of the health researches in five levels: knowledge advanced, capacitating building, impacts on decision-making, impacts on health, and socioeconomic impacts [27–29]. Different studies have put emphasis on this framework’s generalizability in the evaluation of research systems. It seems that payback framework is suitable for evaluating a network’s impacts after it’s establishment.

One major limitation in this study was the differences between the ages of the networks. Based on a network’s years of formation, expectations from the networks with different ages vary (e.g. there are different expectations from networks with 1–3 years, 4–6 years, 7–10 years and beyond 10 years of formation), and evaluation criteria differ for networks with diverse ages [10]. Another limitation was considering the number of articles as the main gauge for the efficiency of knowledge networks, and we ignored other aspects of knowledge networks such as capacity building and their effect on decision making.

Building a powerful innovation system requires powerful knowledge networks. On the other hand, innovation systems are *informal* in developing countries and their progress in technological and organizational infrastructures is unsatisfactory [30]. According to the 2012 Global Innovation Index, Iran has a low rating among countries of upper-middle income class [31]. Therefore in order to achieve the specified goals set forth by the 20-Year-Perspective document, reinforcing the innovation system and embedding knowledge networks into their structure is inevitable.

To develop knowledge and technology in a country, networks need to solve the problems they face in management and governance. That is, as this study concludes, the first step towards the realization of true knowledge networks in Iran’s health system.

## Conclusion

Consolidating and revising policies relevant to the networks and preparing a guide for establishing managing networks could prove helpful. To develop knowledge and technology in a country, networks need to solve the problems they face in management and governance. That is, the first step towards the realization of true knowledge networks in health system.

## Abbreviations

MOHME: Ministry of health and medical education.
